# The Use of Precision‐Cut Lung Slices for Studying Innate Immunity to Viral Infections

**DOI:** 10.1002/cpz1.505

**Published:** 2022-08-08

**Authors:** Christina Michalaki, Charlotte Dean, Cecilia Johansson

**Affiliations:** ^1^ Section of Respiratory Infections, National Heart and Lung Institute Imperial College London London United Kingdom; ^2^ Cardio Respiratory Interface Section, National Heart and Lung Institute Imperial College London London United Kingdom

**Keywords:** cytokines, *ex vivo* model, innate immunity, lower respiratory tract, lung, viral infections

## Abstract

Precision‐cut lung slices (PCLS) are a novel tool to study cells of the lower airways. As PCLS retain the integrity and architecture of the lung, they constitute a robust model for studying the cells of the lower respiratory tract. Use of PCLS for imaging has been previously documented; however, other applications and techniques can also be applied to PCLS to increase their use and therefore decrease the number of animals needed for each experiment. We present a detailed protocol for generating PCLS from the murine lung. We show that cultured PCLS remain viable up to at least 8 days of culture, that RNA can be isolated from the tissue, and that flow cytometry can be carried out on the cells obtained from the PCLS. Furthermore, we demonstrate that cytokines and chemokines can be detected in the culture supernatants of PCLS exposed to viruses. Overall, these protocols expand the use of PCLS, especially for infection studies. © 2022 The Authors. Current Protocols published by Wiley Periodicals LLC.

**Basic Protocol 1**: Precision‐cut lung slices (PCLS)

**Basic Protocol 2**: PCLS culture and viability

**Basic Protocol 3**: RNA isolation from PCLS, cDNA conversion, and RT‐qPCR

**Basic Protocol 4**: Staining of cells from PCLS for flow cytometry

**Basic Protocol 5**: *In vivo* RSV administration and *ex vivo* PCLS RSV exposure

## INTRODUCTION

Respiratory viral infections are a major cause of death worldwide (World Health Organization, 2020). These infections are responsible for one‐quarter of hospitalizations in young children and also cause severe disease in the elderly (Openshaw, Chiu, Culley, & Johansson, 2017; Troy & Bosco, 2016). In the majority of cases, viral infections in the lung are self‐limiting and remain in the upper respiratory tract, causing mild symptoms. However, in susceptible individuals, such as the elderly and infants, the infections can spread to the lower airways, which may cause pneumonia, wheezing, shortness of breath, or bronchiolitis (Troy & Bosco, 2016). Viruses are detected by pattern recognition receptors (PRRs), such as Toll‐like receptors (TLRs), retinoic acid–inducible gene I–like receptors (RIG‐I), and nucleotide‐binding oligomerization domain (NOD)‐like receptors (NLRs), which recognize pathogen‐associated molecular patterns (PAMPs) and other viral molecules, such as RNA produced during the infection process (Brubaker, Bonham, Zanoni, & Kagan, 2015). PRRs are expressed on epithelial and innate immune cells, and their activation upon viral infection leads to the production and release of not only type I and III interferons (IFNs) but also other proinflammatory mediators, such as cytokines and chemokines, which activate the innate and adaptive immune responses and drive the inflammatory response in the lungs (Johansson, 2016; Makris, Paulsen, & Johansson, 2017; Newton, Cardani, & Braciale, 2016; Nuriev & Johansson, 2019; Openshaw et al., 2017). The cellular sources of these mediators and how their induction is regulated are still not fully elucidated, and models to study this in mouse and human lungs are very important for future possible targeting therapies.

Tissue slices were first used in the 1920s to study organ metabolism and toxicology (Warburg & Minami, 1923). In the 1940s, the microtome with a razor blade was developed by Stadie & Riggs (1994), which enabled production of slices with consistent thickness. More advanced microtomes with a vibrating blade are available now, generating slices with more precision and uniform thickness; these slices are known as precision‐cut tissue slices (Gerpe et al., 2018; Liu, Betts, et al., 2019). As the lung tissue has a special structure of alveoli of a honeycomb‐like morphology to allow maximal gas exchange, it is difficult to cut the tissue to obtain precision‐cut lung slices (PCLS). However, in 1987, Placke and Fisher infused the lungs of hamsters and rats with heated agarose, which they then cooled so that the agarose solidified (Placke & Fisher, 1987), allowing the natural inflated state of the lung to be maintained throughout the process of generating PCLS.

As PCLS retain the structural organization and architecture of the lungs, they have been used in lung anatomy studies (Bai et al., 2016; Rosner et al., 2014; Sanderson, 2011). This model has also been used in numerous toxicology studies to assess the efficacy and safety of new therapeutic targets. For example, PCLS from patients with asthma have been used to assess the safety and efficacy of existing and novel targets for asthma treatment (Battram et al., 2006; Bouyssou et al., 2010; Fugazzola, Barton, Niedorf, Kietzmann, & Ohnesorge, 2012; Lötvall et al., 2012; Martin, Uhlig, & Ullrich, 1996). Lung slices have also been utilized to evaluate allergic responses (Cooper et al., 2011), vascular responses (Rieg, Rossaint, Uhlig, & Martin, 2011; Rieg et al., 2014), early fibrosis (Alsafadi et al., 2017), chronic obstructive pulmonary disease (COPD) (Bauer et al., 2010), and alveologenesis (Akram et al., 2019), among other applications.

In addition, PCLS have been used as a model to study a number of respiratory infections, such as infections with adenoviruses (Booth, Coggeshall, Gordon, & Metcalf, 2004), influenza virus (R. Liu et al., 2015; Temann et al., 2017), rhinovirus (Wronski et al., 2021), and respiratory syncytial virus (RSV) (Ebsen et al., 2002). For example, murine PCLS that were exposed to *Chlamydophila pneumoniae* (Cp) and RSV for 1 to 96 hr showed inclusions of both Cp and RSV in different cell types, suggesting that PCLS can be infected by both bacteria and viruses (Ebsen et al., 2002).

PCLS retain *in vivo* tissue integrity and architecture and therefore constitute a robust model for studying viral infections *ex vivo*. To this end, we present a detailed protocol for generating PCLS from the murine lung (Basic Protocol 1). We also show that PCLS can remain viable in culture (Basic Protocol 2), that RNA can be isolated from the tissue for gene expression analysis (Basic Protocol 3), and that flow cytometry can be carried out on cells obtained from the PCLS (Basic Protocol 4). Furthermore, we demonstrate that PCLS can be exposed to viruses *ex vivo* and that subsequently, cytokines and chemokines can be detected in the PCLS culture supernatants (Basic Protocol 5).

*NOTE*: All solutions and equipment coming into contact with cells must be sterile, and proper sterile technique should be used accordingly.

*NOTE*: All culture incubations are performed in a 37°C, 5% CO_2_ incubator unless otherwise specified.

*NOTE*: All protocols involving animals must be reviewed and approved by the appropriate Animal Care and Use Committee and must follow regulations for the care and use of laboratory animals.

## Basic Protocol 1 PRECISION‐CUT LUNG SLICES (PCLS)

This protocol describes a method of generating murine PCLS. It can be used to generate PCLS from other species as well with some modifications.

### Materials

Phosphate‐buffered saline (PBS; Sigma‐Aldrich, cat. no. 806552)
Superglue
Complete DMEM medium (see recipe), 4°C
Adult C57BL/6 mice
Pentobarbital (Centaur)

Vibrating microtome, including metallic clamp and buffer tank (Compresstome® VF‐310‐0Z, Precisionary Instruments)
Blades (World Precision Instruments, cat. no. 7550‐1‐SS)
Blade holder
24‐well plates
50‐ml tubes
1‐ml syringes with 25G needles
Dissection instruments
Cannulas (19G needle with tubing; Intramedic™ Clay Adams Polyethylene Tubing 0.86 × 1.270 mm) connected to 2.5‐ml syringes
Sewing thread
Petri dishes
Disposable scalpels (Swann‐Morton)
Specimen tubes (Precisionary Instruments, cat. no. SKU VF‐SPSK‐VM‐15.5‐BOS)
Forceps
Thin spatula

Additional reagents and equipment for preparing warm (40°C) 1.5% low‐melting‐point agarose (see recipe)

### Slicing preparation

1 Cool PBS to 4°C.
2 Prepare warm (40°C) 1.5% low‐melting‐point agarose.
3 Pre‐cool metallic clamp (from vibrating microtome) at −20°C for ≥20 min prior to slicing. Glue a blade to blade holder using superglue and leave it to set.
4 Pipet 1 ml cold complete DMEM medium into each well of a 24‐well plate and store at 4°C until slicing.
5 Add 35 ml cold complete DMEM medium to a 50‐ml tube and store at 4°C to store lungs after dissection.

### Lung dissection

6 Humanely kill adult C57BL/6 mice with a fatal dose of pentobarbital injected intraperitonially using a 1‐ml syringe with a 25G needle.
7 Remove chest wall using dissection instruments and insert a cannula into the trachea, fixing it using sewing thread.
8 To inflate the adult mouse lungs, slowly inject 1 ml warm (40°C) 1.5% low‐melting‐point agarose (see step 2) into trachea through the cannula connected to a 2.5‐ml syringe.
9 Place ice on top of lungs to allow the agarose to solidify.
10 Once this is achieved, remove lungs in one piece from the chest cavity and keep on ice in cold complete DMEM medium (see step 5) until slicing.

### Precision‐cut lung slicing technique

11 Keep 1.5% low‐melting‐point agarose heated at 40°C (see step 2) and place metallic clamp from step 3 on ice.
12 Place inflated lungs from step 10 into a petri dish filled with cold complete DMEM medium and separate lobes to manageable sizes for the vibrating microtome using a disposable scalpel.
13 Add a small drop of superglue onto end of the plunger of the specimen tube. Pick up a lung lobe using forceps, dab it on a piece of tissue to remove excess medium, and place it onto the superglue.
14 Fill specimen tube with warm (40°C) 1.5% low‐melting‐point agarose (see step 11) until the lung piece is covered and slowly draw plunger into the specimen tube until the agarose is just at the top.
15 Adjust blade holder.
16 Place specimen tube into the slot within the buffer tank in the vibrating microtome securely, until the stopper reaches the base, and fill buffer tank with the cold PBS from step 1.
17 Press forward button on the vibrating microtome until the step motor drive touches the plunger.
18 Adjust advance, oscillation, and thickness to the desired settings using the control box.
19 Start slicing. After discarding the first few smaller PCLS, carefully lift out lung slices using a thin spatula and place them into the 24‐well plate filled with cold complete DMEM medium (see step 4). Once a plate is filled, incubate lung slices in a 37°C, 5% CO_2_ incubator.
20 Continue until reaching end of the lung lobe.
21 Repeat steps 12 through 20 for remaining lung lobes.
  Mice have five lung lobes. Typically, the left lung lobe and the right caudal lobe give the best quality of PCLS. From an adult mouse, around 40 to 50 PCLS can be obtained, depending on how successful the inflation was.

## Basic Protocol 2 PCLS CULTURE AND VIABILITY

The following protocol covers the culturing of murine PCLS and a method to test the viability of the PCLS generated. PCLS generated from different species may require different culturing conditions.

### Materials

PCLS (see Basic Protocol 1)
Complete DMEM medium, 37°C
AlamarBlue dye (Thermo Fisher, cat. no. DAL1025)
Serum‐free DMEM (SF‐DMEM; Sigma‐Aldrich, cat. no. SLM‐020)

24‐well plates
Thin spatula
37°C water bath
96‐well plate
Enzyme‐linked immunosorbent assay (ELISA) plate reader (FLUOstar Omega, BMG Labtech)
MARS software (BMG Labtech)

### PCLS culture preparation

1 Incubate PCLS from Basic Protocol 1 at 37°C, 5% CO_2_ overnight to allow recovery from slicing.
2 The next morning, to remove any excess agarose, add fresh warm complete DMEM medium to new 24‐well plates and move PCLS to the new plate using a thin spatula.
3 Change medium twice by carefully pipetting out the old medium and replacing it with fresh warm medium.
  All washes are performed with 1 ml medium.

### Viability assay

4 Prepare a 10% solution of AlamarBlue dye in SF‐DMEM and pre‐warm in a 37°C water bath.
  Prepare enough dye for all PCLS to be tested.
5 Remove culture supernatant of each PCLS and add 300 μl of the 10% AlamarBlue dye solution to each well. Incubate for 1 hr at 37°C, 5% CO_2_.
6 Transfer supernatant to a 96‐well plate.
7 Add 1 ml fresh warm complete DMEM medium to PCLS and keep them in culture.
8 Measure fluorescence of the supernatant in the 96‐well plate with an ELISA plate reader using 540 nm as the excitation wavelength and 590 nm as the emission wavelength. Analyze data using MARS software.
9 Repeat steps 4 to 8 at any additional time point of interest.

## Basic Protocol 3 RNA ISOLATION FROM PCLS, cDNA CONVERSION, AND RT‐qPCR

In this protocol, an optimized method for RNA isolation from PCLS is described.

### Materials

PCLS (see Basic Protocol 2)
TRIzol (Invitrogen, cat. no. 15596018)
Chloroform
RNeasy Mini kit (Qiagen, cat. no. 74106)
Nuclease‐free water (Promega, cat. no. P1193)
High‐Capacity RNA‐to‐cDNA kit (Thermo Fisher, cat. no. 4387406)
Quantitect Probe PCR Master Mix (Qiagen, cat. no. 204345)
Target primers (see Table 1)

**Table 1:** Target Primers

Primer	Company	Cat. no.
*Gapdh*	Applied Biosystems	4352339E
*Actb*	Applied Biosystems	Mm00607939_s1
*Hprt*	Applied Biosystems	Mm00446968_m1

1.5‐ml Eppendorf tubes
Tissue homogenizer (TissueLyser LT, Qiagen)
Gel Eppendorf tubes (VWR, cat. no. 733‐2478)
Refrigerated microcentrifuge, 4°C
Spectrophotometer (NanoDrop 1000 Spectrophotometer, Thermo Scientific)
96‐well PCR plate (MicroAmp™ Optical 96‐Well Reaction Plate, Applied Biosystems, cat no. N8010560)
7500 Fast Real‐Time PCR System (Applied Biosystems)
7500 Fast System SDS Software (Applied Biosystems)

### RNA isolation

1 For every different condition, pool PCLS that have received the same stimulus in a 1.5‐ml Eppendorf tube containing 350 μl TRIzol and homogenize using a tissue homogenizer. Store slices at −80°C for later RNA isolation.
2 Briefly pre‐spin gel Eppendorf tubes to collect gel at the bottom of the tubes.
3 Add samples from step 1 to the gel Eppendorf tubes and incubate for 5 min at room temperature.
4 Add 200 μl chloroform to each tube and then manually shake vigorously for 15 s (do not vortex).
5 Centrifuge samples for 10 min at 12,000 × *g*, 4°C.
6 Pour aqueous phase of the sample into a new 1.5‐ml Eppendorf tube.
7 Extract RNA using RNeasy Mini kit, including the optional DNA digestion step as per the manufacturer's instructions. Elute RNA in 30 μl nuclease‐free water.
8 Determine RNA concentration using a spectrophotometer and then convert 0.25 or 0.5 μg RNA to cDNA using the High‐Capacity RNA‐to‐cDNA kit according to the manufacturer's instructions.

### RT‐qPCR

9 Prepare a mixture of the Quantitect Probe PCR Master Mix, the target primers (see Table 1), and nuclease‐free water according to the manufacturer's instructions.
10 Pipet 11.5 μl mixture into each well of a 96‐well PCR plate and add 1 μl of each cDNA sample from step 8. Run each sample in duplicate.
11 Use 7500 Fast Real‐Time PCR System to perform RT‐qPCR according to the manufacturer's instructions.
12 Perform analysis using 7500 Fast System SDS Software.
13 To quantify relative mRNA expression, calculate mean ΔCt for each target gene relative to *Gapdh* and express as 2^−ΔCt^.

## Basic Protocol 4 STAINING OF CELLS FROM PCLS FOR FLOW CYTOMETRY

The protocol below describes the digestion of PCLS to obtain cells to stain them for markers used to mainly identify immune, epithelial, and endothelial cells using flow cytometry.

### Materials

2 mg/ml collagenase D (see recipe) or 5 mg/ml dispase II (see recipe)
10 mg/ml DNase I from bovine pancreas (Roche, cat. no. 10104159001)
PCLS (see Basic Protocol 2)
Complete DMEM medium (see recipe), 37°C
PBS (Sigma‐Aldrich, cat. no. 806552), room temperature and 4°C
0.4% (w/v) trypan blue (Sigma‐Aldrich, cat. no. T8154)
FACS buffer (see recipe), 4°C
Fc block (rat IgG2b anti‐mouse CD16/CD32 receptor antibody; BD Pharmingen™, cat. no. 553141) diluted 1:200 in FACS buffer (see recipe), 4°C
Surface stain antibody mix (see Table 2) in PBS (Sigma‐Aldrich, cat. no. 806552), 4°C
Cytofix™ fixation buffer (BioLegend, cat. no. 420801), 4°C

**Table 2:** Antibodies Used for Flow Cytometry Staining

Surface stain	Conjugate	Clone	Dilution	Company	Cat. no.
L/D	Aqua	N/A	1:1000	Invitrogen	L34957
CD11b	AF700	M1/70	1:400	Invitrogen	56‐0112‐82
CD11c	APC	N418	1:400	BioLegend	117310
CD45	BV605	30‐F11	1:400	BioLegend	103140
Siglec‐F	BV786	E50‐2440	1:400	BD Biosciences	740956
CD326 (EpCAM)	FITC	G8.8	1:200	Invitrogen	11‐5791‐80
CD31	PE	MEC13.3	1:200	BioLegend	102508
CD103	PerCP‐ cyanine5.5	2E7	1:100	BioLegend	121416
CD26	BV711	H194‐112	1:100	BD Biosciences	740678

15‐ml Falcon tubes
Shaker (optional)
Refrigerated centrifuge, room temperature and 4°C
100‐μm cell strainers (Greiner Bio‐One, cat. no. 542000)
Counting slides (Immune Systems, cat. no. BVS100)
Light microscope
96‐well plate

1 In a 15‐ml Falcon tube, combine 1.5 ml of 2 mg/ml collagenase D or 5 mg/ml dispase II and 12 μl of 10 mg/ml DNase I from bovine pancreas. Pool six PCLS into this tube and incubate for 45 min in a 37°C, 5% CO_2_ incubator (with shaking if possible).
2 Add 1.5 ml warm complete DMEM medium.
3 Pipet up and down until slices are dissolved.
4 Top up with 3 ml PBS.
5 Centrifuge 10 min at 200 × *g* and remove supernatant.
6 Add 5 ml PBS to tube.
7 Filter cells through a 100‐μm cell strainer and into a new 15‐ml Falcon tube to obtain a single‐cell suspension. Centrifuge 10 min at 200 × *g* and remove supernatant.
8 Resuspend in 500 μl PBS and count cells on counting slides under a light microscope using 0.4% trypan blue at 1:4.
9 Transfer 1 × 10^6^ lung cells into each well of a 96‐well plate. Plate cells for an unstained control and heat‐kill some cells for a live/dead sample.
10 Centrifuge 10 min at 200 × *g*, 4°C, and remove supernatant.
11 Wash plate with 200 μl cold FACS buffer per well and spin again as in step 10.
12 Add 50 μl Fc block diluted 1:200 in cold FACS buffer per well and incubate plate for 20 min at 4°C.
13 Add 100 μl cold PBS per well and spin again as in step 10.
14 Add 50 μl surface stain antibody mix (see Table 2) in cold PBS for surface staining and live/dead assessment. Incubate for 30 min at 4°C.
15 Add 200 μl cold FACS buffer per well and spin as in step 10.
16 Add 100 μl cold Cytofix™ fixation buffer to all wells. Incubate for 20 min at 4°C.
17 Top up with 100 μl cold FACS buffer per well and spin as in step 10.
18 Add 200 μl cold FACS buffer per well and spin as in step 10.
19 Add 150 μl cold FACS buffer per well and leave plate overnight at 4°C before proceeding to flow cytometry.

## Basic Protocol 5 *IN VIVO* RSV ADMINISTRATION AND *EX VIVO* PCLS RSV EXPOSURE

This protocol covers *in vivo* RSV administration and *ex vivo* RSV exposure of murine PCLS.

### Materials

PCLS (see Basic Protocol 1)
RSV (originally A2 strain from ATCC)
Complete DMEM medium (see recipe), 37°C
Adult C57BL/6 mice
PBS (Sigma‐Aldrich, cat. no. 806552)
Pentobarbital (Centaur)
DuoSet ELISA kit (R&D Systems)

1‐ml syringes with 25G needles
ELISA plate reader (FLUOstar Omega, BMG Labtech)
MARS software (BMG Labtech)

Additional reagents and equipment for PCLS homogenization and RNA extraction (see Basic Protocol 3, steps 1 to 8), mouse anesthesia (see Current Protocols article: Davis, 2008), and lung removal and PCLS processing (see Basic Protocol 1, steps 7 to 21)

*CAUTION*: RSV is a Biosafety Level 2 (BSL‐2) pathogen. Follow all appropriate guidelines and regulations for the use and handling of pathogenic microorganisms.

*NOTE*: Plaque‐purified human RSV is grown in HEp2 cells (Lee et al., 2010).

### Ex vivo RSV exposure of PCLS

1a Expose PCLS to different concentrations [5 × 10^3^, 5 × 10^4^, or 5 × 10^5^ focus‐forming units (FFUs)] of RSV in warm complete DMEM medium or medium control in a total volume of 200 μl per well.
2a Incubate PCLS at in a 37°C, 5% CO_2_ incubator for 2 hr.
3a Top up with 300 μl warm complete DMEM medium.
4a Incubate PCLS for the desired time.
5a Collect supernatants for ELISA (see steps 6 and 7). Pool PCLS, homogenize in 350 μl TRIzol, and perform RNA extraction (see Basic Protocol 3, steps 1 to 8).

### In vivo RSV infection and PCLS culture

1b For *in vivo* infection, anesthetize adult C57BL/6 mice prior to intranasal administration of 100 μl containing desired concentration of RSV or PBS control.
2b Sacrifice mice 2 hr post‐infection with a fatal dose of pentobarbital injected intraperitonially using a 1‐ml syringe with a 25G needle.
3b Remove lungs of the mice and subsequently process to obtain PCLS as described in Basic Protocol 1, steps 7 to 21.

### Viral load determination

6 For RSV load, calculate exact number of copies of the RSV L gene using a plasmid DNA standard curve. Normalize results using *Gapdh*, as described in Goritzka et al. (2014).

### Immune mediator detection

7 Perform ELISA to measure the concentration of CXCL1 (using the DuoSet ELISA kit) and IL‐6 [using the ELISA described in Goritzka et al. (2014)].
8 Determine absorbance at 450 nm with an ELISA plate reader and analyze data using MARS software.

## REAGENTS AND SOLUTIONS

### Collagenase D, 2 mg/ml

Dilute collagenase D (Sigma‐Aldrich, cat. no. 11088866001) to 2 mg/ml in sterile 10% (v/v) FBS (Gibco) in sterile PBS (Sigma‐Aldrich, cat. no. 806552). Store overnight at −20°C. Bring to room temperature before use.

### Complete DMEM medium

Supplement Dulbecco's modified Eagle's medium (DMEM; Sigma‐Aldrich, cat. no. SLM‐020) with 1× penicillin‐streptomycin (from 100×; Gibco, cat. no. 10378016) and 1× L‐glutamine (from 100×; Gibco, cat. no. 25030081). In some experiments, supplement medium with 10% (v/v) heat‐inactivated fetal bovine serum (Gibco). Store ≤1 month at 4°C.

### Dispase II, 5 mg/ml

Dilute dispase II (Sigma‐Aldrich, cat. no. 4942078001) to 5 mg/ml in sterile PBS (Sigma‐Aldrich, cat. no. 806552). Store ≤1 month at −20°C.

### FACS buffer

Supplement PBS (Sigma‐Aldrich, cat. no. 806552) with 1% (w/v) bovine serum albumin (BSA; Sigma‐Aldrich, cat. no. A7906) and 5 mM EDTA (Thermo Fisher, cat. no. 15575020). Sterile‐filter and store ≤1 month at 4°C.

### Low‐melting‐point agarose, 1.5%

Prepare 1.5% (w/v) low‐melting‐point agarose (Sigma‐Aldrich, cat. no. A9539) in PBS (Sigma‐Aldrich, cat. no. 806552). Heat solution in the microwave for 30 s twice until it becomes clear and store it in a 40°C water bath before use to prevent it from solidifying. Prepare fresh immediately before use.

## COMMENTARY

### Background Information

PCLS retain the complex architecture of the lung as they retain the small airways, the respiratory parenchyma, and the various pulmonary cell types (Liu, Betts, et al. 2019). Therefore, PCLS are a popular model, especially for toxicological and pharmaceutical research. However, PCLS can also be used for other applications where the cells of the lower respiratory tract are studied. One advantage of the model is that it is cost effective, as many PCLS can be prepared from one organ and be used to test a variety of different experimental conditions (Majorova et al., 2021). A deficit of the model is the absence of blood flow, which renders the PCLS unable to recruit immune cells. Despite the lack of active circulation, slices have been used as a model to study idiopathic pulmonary fibrosis, asthma, COPD, allergy, and infections (Alsafadi et al., 2017; Cooper et al., 2011; Dijk, Culha, Menzen, Bidan, & Gosens, 2016; Liu, Henry, et al., 2019; Wu, van Dijk, Bos, Kistemaker, & Gosens, 2019). We have developed several protocols for immunology research using PCLS and viral infections, and we present experimental methods that can be used in this model to study gene expression, protein production, and specific cell types.

### Critical Parameters and Troubleshooting

Extra attention should be given to the thickness of the PCLS (Basic Protocol 1) and how this influences the experimental use and readout of the slices (Table 3). Therefore, it is important to conduct preliminary experiments with different‐thickness PCLS so that the optimal thickness of the tissue for its application can be determined. The thickness has to remain consistent between experiments so that the results can be comparable. Another important factor to take into consideration is that low‐melting‐point agarose should be used to inflate the lungs. This is because the temperature of the agarose needs to be lower than the body temperature without gelling (Sanderson, 2011). The percentage of the agarose used also plays an important role, as it can affect the mechanical properties of the tissue. Typically, lower percentages of agarose are used for filling rodent tissue, and higher percentages are used for tissues of larger animals and humans (Alsafadi et al., 2017). For Basic Protocol 3, it is important to elute the RNA in 30 μl nuclease‐free water, as the RNA concentration obtained from the PCLS is not high. For Basic Protocol 4, the enzyme used to digest the PCLS should be carefully chosen and tested depending on the cells of interest.

**Table 3:** Troubleshooting Guide for Using Murine PCLS

Problem	Possible cause	Solution
Inconsistent thickness	Vibratome calibration issue	Do not collect PCLS that seem thicker than expected. Wait for the machine to recalibrate.
High background levels of cytokine and chemokine production	Addition of FBS to culture medium	Do not add FBS to the culture medium.
Low RNA concentration and quality	Agarose in the tissue	Perform multiple washes to remove excess agarose. Pool PCLS to increase RNA yield. Elute in the lowest recommended volume of nuclease‐free water.

### Understanding Results

**Figure 1:**
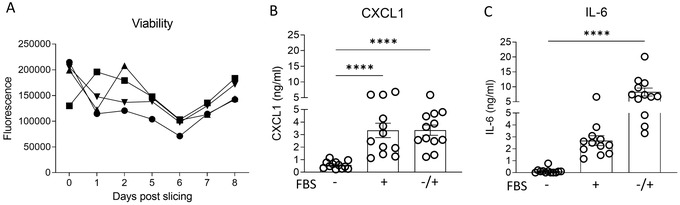
PCLS remain viable in culture for ≥8 days, and medium supplemented with FBS increases background expression of CXCL1 and IL‐6. (**A**) Viability of unstimulated mouse PCLS, expressed as fluorescence using AlamarBlue dye. Individual slices are represented with different symbols. One experiment, n = 4. Individual slices are shown. Protein expression of (**B**) CXCL1 or (**C**) IL‐6 in culture supernatants of PCLS at 24 hr after culturing in serum‐free medium, in medium supplemented with FBS, or in serum‐free medium overnight and then serum‐supplemented medium for 24 hr, as measured by ELISA. Data are shown as mean ± SEM. Each dot represents one PCLS, three independent experiments pooled, n = 12. One‐way ANOVA + Dunnett's multiple comparison test; * indicates a significant difference between serum‐free medium and medium supplemented with FBS, **** *p* < .0001.

Murine PCLS can be kept in culture (Basic Protocol 2) for several days. Figure 1A illustrates that the viability of individual slices stays the same during 8 days of culture. However, the culture conditions can influence the performance of the PCLS. For example, use of FBS‐supplemented medium to culture slices has been controversial in the literature (Tables 3 and 4). Addition of FBS to the medium could cause fibroblast proliferation and therefore influence the experimental readouts. Therefore, we tested whether FBS‐supplemented medium compared to serum‐free medium had an effect on the secretion of chemokines (e.g., CXCL1) and cytokines (e.g., IL‐6) from slices. To perform these experiments, a group of PCLS was cultured in SF‐DMEM (supplemented with penicillin, streptomycin, and L‐glutamine but no FBS), a second group of PCLS was cultured in FBS‐supplemented medium, and a third group of PCLS was cultured in SF‐DMEM overnight immediately after slicing and then in FBS‐supplemented medium for 24 hr. The supernatants of all the groups were collected for ELISA analysis. As shown in Figures 1B and C, the addition of FBS to the medium has an effect on both IL‐6 and CXCL1 detected in the culture supernatants of PCLS and increases the production significantly, which can alter the results after PCLS have been stimulated with, for example, viruses. From Table 4, it is evident that most research groups cultured their PCLS in serum‐free medium. In a study by Bryson et al. (2020), the viability of avian PCLS was assessed over a period of 7 days in different culturing medium and growth factors. It was shown that slices cultured in the absence of FBS had a 30% higher cell viability compared to slices cultured in FBS‐rich medium. The number of dead cells was also 38% greater in PCLS cultured in medium with FBS, suggesting that FBS has a detrimental effect on tissue viability (Bryson et al., 2020). It is therefore important to consider the culture conditions and, for most assays, to avoid addition of FBS to the culture.

**Table 4:** Summary of Culture Conditions (With or Without FBS) for PCLS

Species	Culture medium type^a^	Reference
Murine	SF	Akram et al., 2019
Avian	SF	Bryson et al., 2020
Human	SF	Temann et al., 2017
Human and murine	With FBS	Bailey et al., 2020
Murine	SF	Wu et al., 2019
Human	SF	Lauenstein et al., 2014
Human	SF	Temann et al., 2017
Murine	SF	Henjakovic et al., 2008
Murine	SF	Ebsen et al., 2002
Murine, rat, human, and non‐ human primate	With FBS	Niehof et al., 2017
Human	SF	Sun et al., 2015
Murine	SF	Hiorns et al., 2016
Murine	With FBS	Puttur et al., 2019
Human	SF	Woodcock et al., 2018
Guinea pig	SF	Ressmeyer et al., 2006
Rat	SF	Hirn et al., 2014
Human	With FBS	Banerjee, Huckuntod, Mills, Kurten, & Pechous, 2019
Rat	SF	Huang et al., 2019
Bovine and caprine	Both SF and with FBS	Weldearegay et al., 2019

a SF, serum‐free medium. With FBS, medium supplemented with fetal bovine serum.

**Figure 2:**
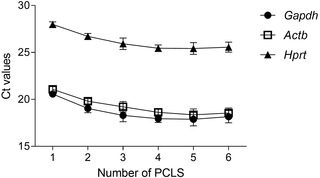
*Gapdh* and *Actb* are robust housekeeping genes for PCLS. RNA isolated from different numbers of PCLS was converted to cDNA, and the Ct values for the expression of the housekeeping genes *Hprt*, *Actb*, and *Gapdh* are shown. Data are shown as mean ± SEM. Three independent experiments pooled, n = 3.

The ability to extract RNA and perform gene expression analysis (see Basic Protocol 3) broadens the applications for this model. However, RNA extraction from PCLS can be challenging due to the high concentration of agarose present in the tissue (Table 3). As shown in Table 5, RNA was isolated from different numbers of slices following Basic Protocol 3. The experiment was repeated three times, and the average RNA concentration is specified (Table 5). Using one PCLS per tube yielded very small quantities of RNA for conversion to cDNA and subsequent RT‐qPCR. Four PCLS seems to be the ideal number for extracting a high enough RNA concentration. Surprisingly, five and six PCLS yields similar or lower concentrations of RNA, respectively, compared to four PCLS per tube. This might be due to the agarose content, which increases with more PCLS pooled. In another study by Niehof et al. (2017), RNA extraction from two mouse PCLS resulted in similar levels of RNA; however, a different protocol was used, with additional cleanup steps that might have led to a greater concentration of RNA (Niehof et al., 2017). The quality and purity of the RNA, as observed in Table 6, correlates to the amount of RNA extracted, and better 260/280 and 260/230 ratios are obtained when pooling four PCLS or more. Of note, for certain applications, this degree of purity might not be sufficient. Following conversion of the RNA obtained to cDNA, RT‐qPCR was performed to detect the housekeeping genes *Hprt*, *Actb*, and *Gapdh*. All housekeeping genes were detected, but the best (lowest Ct) was detected in the samples pooling four or more PCLS for RNA extraction (Fig. 2). *Hprt* showed a relatively high Ct, suggesting that use of *Gapdh* or *Actb* as a housekeeping gene is preferable.

**Table 5:** Concentration of RNA from PCLS from Three Different Experiments^a^

	Number of PCLS	1	2	3	4	5	6
**Experiment 1**	Concentration (ng/μl)	30.1	129.3	84.7	71.3	89.2	83.3
**Experiment 2**	Concentration (ng/μl)	47.5	59.2	78.3	92.1	115.2	76.1
**Experiment 3**	Concentration (ng/μl)	19.9	54.2	24.9	68.0	48.7	72.4
**Average**	Concentration (ng/μl) ± SD	32.5 ± 14.0	80.9 ± 42.0	62.6 ± 32.8	77.1 ± 13.1	84.4 ± 33.5	77.3 ± 5.5

a Data are shown as mean ± SD. Three independent experiments are shown.

**Table 6:** 260/280 and 260/230 Absorbance of RNA from PCLS^a^

	Number of PCLS	1	2	3	4	5	6
**Experiment 1**	Absorbance 260/280	1.57	1.53	1.66	1.85	1.99	1.94
	Absorbance 260/230	0.65	0.69	0.72	1.13	1.49	0.74
**Experiment 2**	Absorbance 260/230	1.54	1.59	1.71	1.77	1.67	1.66
	Absorbance 260/230	0.31	0.58	0.51	0.94	0.87	0.74
**Experiment 3**	Absorbance 260/230	1.40	1.52	1.57	1.56	1.59	1.58
	Absorbance 260/230	0.74	0.66	0.73	0.71	0.83	0.74

a Three independent experiments are shown.

**Figure 3:**
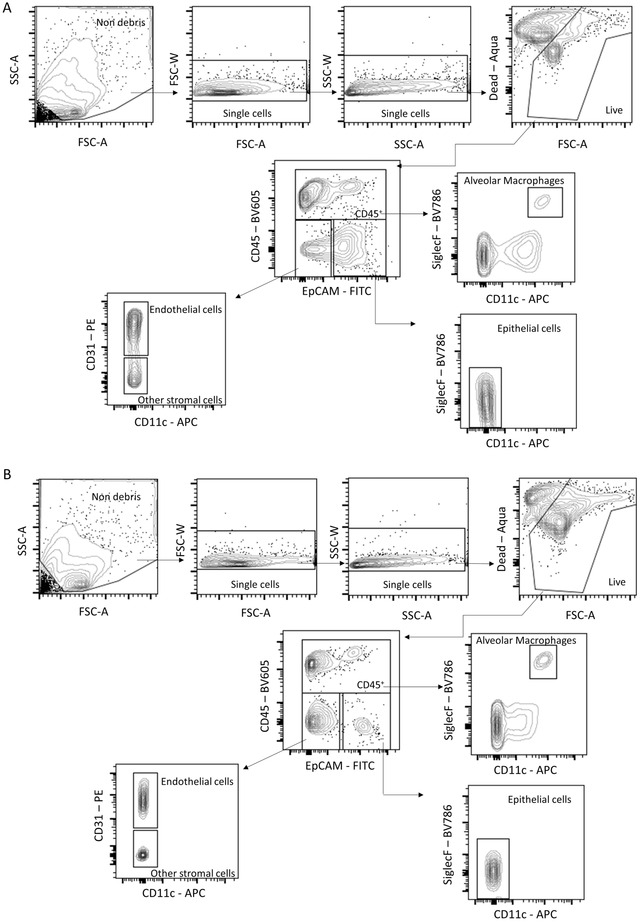
Gating strategy for identifying cell populations in PCLS. (**A**) PCLS were generated and digested with collagenase to obtain lung cells, which were stained for the indicated cell surface molecules. After excluding debris and after gating on singlet live cells, the depicted gates were used to identify CD45^+^ cells and CD45‐ cells to identify alveolar macrophages, endothelial cells, other stromal cells, and epithelial cells. (**B**) PCLS were generated and digested with dispase to obtain lung cells, which were stained for the indicated cell surface molecules. After excluding debris and after gating on singlet live cells, the depicted gates were used to identify CD45^+^ cells and CD45‐ cells to identify alveolar macrophages, endothelial cells, other stromal cells, and epithelial cells.

**Figure 4:**
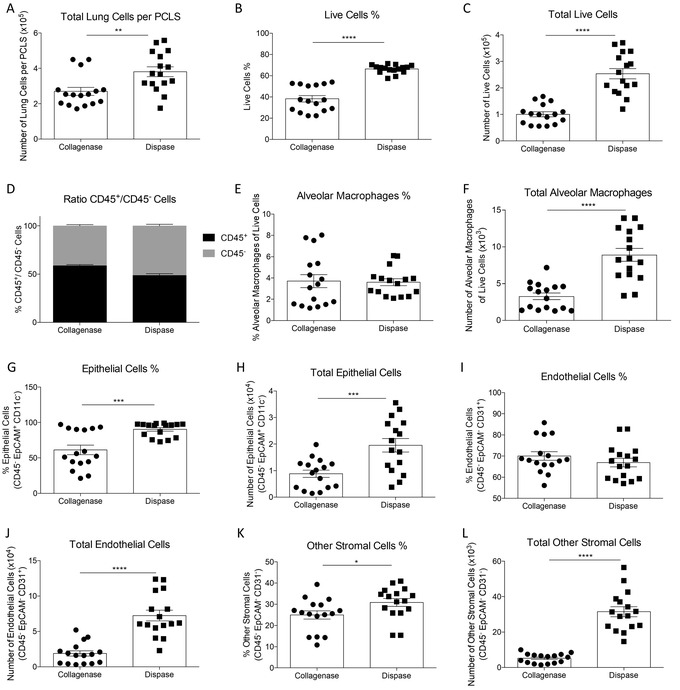
Types of cells identified in collagenase‐ and dispase‐digested PCLS. (**A**) Total number of lung cells obtained from collagenase‐ and dispase‐digested PCLS. Percentage (**B**) and total number (**C**) of live cells obtained from collagenase‐ and dispase‐digested PCLS. (**D**) Ratio of CD45+ to CD45‐ cells obtained from collagenase‐ and dispase‐digested PCLS. (**E**) Percentage and (**F**) total number of alveolar macrophages and (**G**) percentage and (**H**) total number of epithelial cells obtained from collagenase‐ and dispase‐digested PCLS. (**I**) Percentage and (**J**) total number of endothelial cells and (**K**) percentage and (**L**) total number of other stromal cells obtained from collagenase‐ and dispase‐digested PCLS. Data are shown as mean ± SEM. Each dot represents six PCLS digested to obtain lung cells, three independent experiments pooled, n = 16. Unpaired Student's *t* test; * indicates a significant difference between collagenase‐ and dispase‐digested PCLS, *p < .05; **, *p* < .01; ***, *p* < .001, **** *p* < .0001.

Another useful application is to perform flow cytometry analysis of cells obtained from PCLS (Basic Protocol 4). We tried two protocols for digestion of the tissue, using the digesting enzyme collagenase or dispase. Single‐cell suspensions were then stained for markers to identify lung cell populations (gating strategy shown in Fig. 3). Overall, we obtained better cell recovery after dispase treatment, with more total and live cells detected (Fig. 4A to 4C). Additionally, more epithelial cells survived when PCLS were digested with dispase compared to collagenase digestion. In the PCLS digested with dispase, the total number of alveolar macrophages was significantly higher. Dispase‐digested slices also had a higher percentage and number of epithelial cells, endothelial cells, and other stromal cells (Fig. 4D to 4L).

**Figure 5:**
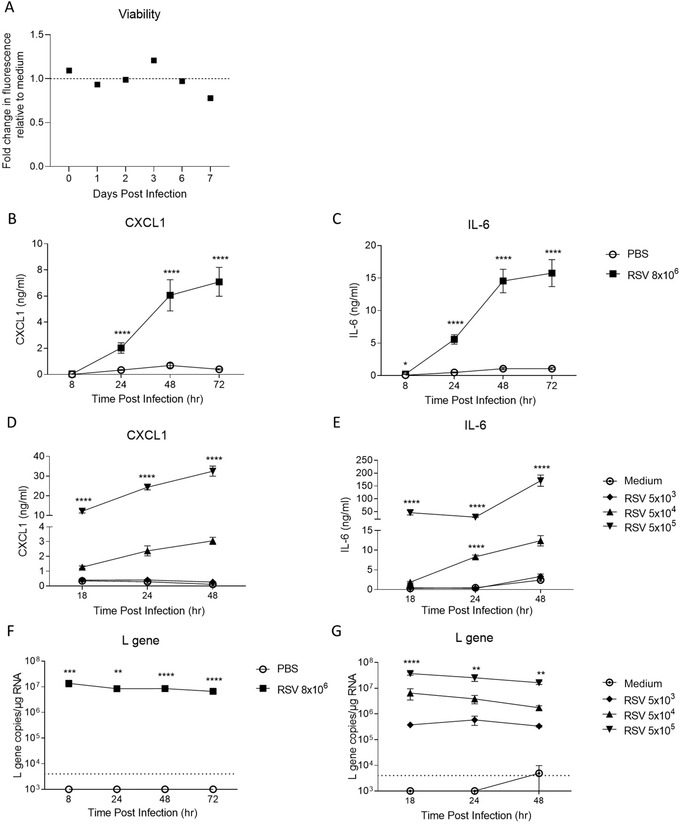
*In vivo* RSV infection of mice before generating PCLS and *ex vivo* RSV exposure of naïve PCLS produce similar cytokine and chemokine responses and L gene expression. (**A**) Viability of mouse PCLS exposed to RSV *ex vivo*, expressed as the fold change in fluorescence relative to the medium control (shown by the dashed line) using AlamarBlue dye up to 7 days post‐infection. PCLS were obtained from mice infected with 8 × 10^6^ FFUs RSV and kept in culture for 8 hr, 24 hr, 48 hr, or 72 hr, or PCLS from naïve mice were exposed to 5 × 10^3^, 5 × 10^4^, or 5 × 10^5^ FFUs RSV and kept in culture for 18 hr, 24 hr, or 48 hr. Two experiments pooled, n = 2. Protein expression of (**B**) CXCL1 or (**C**) IL‐6 in culture supernatants of *in vivo* RSV‐infected PCLS. Data are presented as mean ± SEM of three mice per group and are representative of three independent experiments, n = 12. Protein expression of (**D**) CXCL1 or (**E**) IL‐6 in culture supernatants of *ex vivo* RSV‐exposed PCLS. Data are presented as mean ± SEM and are representative of at least three independent experiments, n = 12. (**F**) Viral L gene copies were quantified by RT‐qPCR in PCLS obtained from intranasally infected mice as in (B) and (C) at 8 hr, 24 hr, 48 hr, and 72 hr post‐infection. (**G**) Viral L gene copies were quantified by RT‐qPCR in PCLS exposed to RSV as in (D) and (E) at 18 hr, 24 hr, and 48 hr post‐infection. Data are presented as mean ± SEM and are representative of at least three independent experiments, n = 6. One‐way ANOVA + Dunnett's multiple comparison test; * indicates a significant difference between PBS and RSV groups or between medium and RSV at 5 × 10^3^, 5 × 10^4^, or 5 × 10^5^ FFUs. *p < .05, **, *p* < .01; ***, *p* < .001 **** *p* < .0001. Dotted line = limit of detection.

Finally, the ability to perform viral infection studies using PCLS (Basic Protocol 5) is a useful tool. Firstly, the viability of PCLS infected with RSV was compared to that of PCLS kept in non‐RSV medium, and there was no noticeable difference (Fig. 5A). We then wanted to compare how PCLS from lung tissue after *in vivo* RSV infection compared to naïve PCLS exposed to RSV *ex vivo*. Mice were infected with RSV intranasally, and 2 hr later, the lungs were used to obtain PCLS. These slices were compared to PCLS generated from naïve mouse lung tissue exposed to RSV *ex vivo*, and the production of CXCL1 and IL‐6 was quantified in the PCLS supernatant. Both *in vivo* RSV‐infected and *ex vivo* RSV‐exposed PCLS produced CXCL1 and IL‐6, and this production increased significantly over time in culture compared to PBS‐ or medium‐exposed PCLS (Fig. 5B to 5E). In addition, when comparing the viral load (L gene expression), a similar trend was observed in the *in vivo* RSV‐infected and *ex vivo* RSV‐exposed PCLS, and a significantly higher amount of RSV L gene copies was detected at all time points compared to PBS and medium controls (Fig. 5F and 5G).

Taken together, these findings show that mouse PCLS can be used for gene expression and flow cytometry analyses, providing a valid model to study chemokine and cytokine production from cells of the lower respiratory tract after *ex vivo* respiratory viral infections.

### Time Considerations

Depending on the user's experience and the numbers of mice and PCLS needed for the experiments, slicing (Basic Protocol 1) can take ≥1 hr. Preparation of the reagents and the vibratome takes 30 min. Regarding culturing of the slices (Basic Protocol 2), washing of 24 slices (one slice per well in a 24‐well plate) usually takes ≥30 min, so the number of plates to be washed has to be considered. For the viability assay (Basic Protocol 2), the 1‐hr incubation should be taken into account. The RNA extraction along with the cDNA conversion (Basic Protocol 3) can take 3 to 4 hr, depending on the user's experience with the technique. The flow cytometry staining preparation (Basic Protocol 4) usually takes 5 to 6 hr, depending on the compound used for the digestion and the centrifugation times. For the *in vivo* and the *ex vivo* virus exposures (Basic Protocol 5), the 2‐hr incubation time needs to be considered.

### Author Contributions

**Christina Michalaki**: Formal analysis, Investigation, Methodology, Visualization, Writing — original draft; **Charlotte Dean**: Methodology, Writing — review and editing; **Cecilia Johansson**: Conceptualization, Data curation, Funding acquisition, Project administration, Supervision, Validation, Writing — original draft.

### Conflict of Interest

No conflict of interest has been identified.

## Data Availability

The data, tools, and materials (or their source) that support the protocols are available from the corresponding author upon reasonable request.
